# Clinical and Radiographic Factors Do Not Accurately Diagnose Smear-Negative Tuberculosis in HIV-infected Inpatients in Uganda: A Cross-Sectional Study

**DOI:** 10.1371/journal.pone.0009859

**Published:** 2010-03-26

**Authors:** J. Lucian Davis, William Worodria, Harriet Kisembo, John Z. Metcalfe, Adithya Cattamanchi, Michael Kawooya, Rachel Kyeyune, Saskia den Boon, Krista Powell, Richard Okello, Samuel Yoo, Laurence Huang

**Affiliations:** 1 Division of Pulmonary and Critical Care Medicine, San Francisco General Hospital, University of California San Francisco, San Francisco, California, United States of America; 2 Division of HIV/AIDS, San Francisco General Hospital, University of California San Francisco, San Francisco, California, United States of America; 3 Department of Medicine, San Francisco General Hospital, University of California San Francisco, San Francisco, California, United States of America; 4 Francis J. Curry National Tuberculosis Center, San Francisco General Hospital, University of California San Francisco, San Francisco, California, United States of America; 5 Makerere University-University of California San Francisco Research Collaboration, Mulago Hospital, Makerere University, Kampala, Uganda; 6 Department of Medicine, Mulago Hospital, Makerere University, Kampala, Uganda; 7 Department of Radiology, Mulago Hospital, Makerere University, Kampala, Uganda; MRC Laboratories, Gambia

## Abstract

**Background:**

Although World Health Organization guidelines recommend clinical judgment and chest radiography for diagnosing tuberculosis in HIV-infected adults with unexplained cough and negative sputum smears for acid-fast bacilli, the diagnostic performance of this approach is unknown. Therefore, we sought to assess the accuracy of symptoms, physical signs, and radiographic findings for diagnosing tuberculosis in this population in a low-income country with a high incidence of tuberculosis.

**Methodology:**

We performed a cross-sectional study enrolling consecutive HIV-infected inpatients with unexplained cough and negative sputum smears for acid-fast bacilli at Mulago Hospital in Kampala, Uganda. Trained medical officers prospectively collected data on standard symptoms and signs of systemic respiratory illness, and two radiologists interpreted chest radiographs in a standardized fashion. We calculated positive- and negative-likelihood ratios of these factors for diagnosing pulmonary tuberculosis (defined when mycobacterial cultures of sputum or bronchoalveolar lavage fluid were positive). We used both conventional and novel regression techniques to develop multivariable prediction models for pulmonary tuberculosis.

**Principal Findings:**

Among 202 enrolled HIV-infected adults with negative sputum smears for acid-fast bacilli, 72 (36%) had culture-positive pulmonary tuberculosis. No single factor, including respiratory symptoms, physical findings, CD4+ T-cell count, or chest radiographic abnormalities, substantially increased or decreased the likelihood of pulmonary tuberculosis. After exhaustive testing, we were also unable to identify any combination of factors which reliably predicted bacteriologically confirmed tuberculosis.

**Conclusions and Significance:**

Clinical and radiographic criteria did not help diagnose smear-negative pulmonary tuberculosis among HIV-infected patients with unexplained cough in a low-income setting. Enhanced diagnostic methods for smear-negative tuberculosis are urgently needed.

## Introduction

The lack of accurate, rapid, inexpensive tests for the diagnosis of pulmonary tuberculosis (TB) remains a major obstacle to effective TB control, especially in high-burden countries in sub-Saharan Africa where HIV co-infection is common. Sputum smear microscopy, the standard diagnostic test for TB in low-income countries, fails to diagnose about one-third to one-half of all TB patients in systematic reviews. [1] Therefore, clinicians must either use individual judgment to decide whether to treat empirically for TB or to refer to a higher level of care without treatment. Although both approaches are sanctioned by major international guidelines [2], [3], there are limited data on how well clinical and radiographic factors perform for TB diagnosis in HIV-infected patients relative to established gold standards such as mycobacterial culture. [4], [5] Since existing data may not apply to the referral hospital setting envisaged in guidelines, we designed a diagnostic cross-sectional study with two-month follow-up of HIV-infected Ugandan adults with unexplained cough and negative sputum smears for acid-fast bacilli (AFB). The objective of this study was to identify routinely available clinical and/or radiographic predictors or combinations of predictors with a strong likelihood of diagnosing or excluding pulmonary TB.

## Methods

### Ethics Statement

The Makerere University Faculty of Medicine Research and Ethics Committee, the Mulago Hospital Institutional Review Board, the Committee on Human Research at the University of California, San Francisco, and the Uganda National Council for Science and Technology approved the protocol. Some of these patients have been previously included in published studies. [13], [14], [15]

### Participants

During the period September 17, 2007–July 16, 2008, we prospectively enrolled consecutive HIV-infected adult patients admitted to the Medical Emergency Ward at Mulago National Referral Hospital in Kampala, Uganda, with cough of ≥2 weeks but <6 months duration. We excluded patients with a history of TB treatment in the previous two years, as well as those already receiving anti-TB treatment. Each patient provided two sputum samples for direct Ziehl-Neelsen light microscopy and Lowenstein-Jensen mycobacterial culture, after a laboratory technician provided standardized instructions on proper sputum submission. [6] For this analysis, we excluded patients with a positive sputum AFB smear.

### Procedures

After written informed consent, a single medical officer (RK) obtained demographic and clinical information from participants in a standardized interview. The medical officer referred patients for frontal chest radiography according to the hospital's standard protocol for evaluating patients suspected of TB, and digitally photographed the films. Two board-certified radiologists (HK, MK) who were blinded to all clinical and laboratory data reviewed the digitized images according to a standardized interpretation form, which was based on the previously validated Chest Radiographic Reviewing and Reporting System. [7], [8] The readers involved a third radiologist (RO) to adjudicate whenever there were differences in interpretation, and the differences were resolved by consensus.

The study team referred patients for bronchoscopy with bronchoalveolar lavage (BAL), which clinical investigators (WW, SY, AC, JLD) performed according to a standardized protocol that included airway inspection for Kaposi's sarcoma lesions and collection of BAL fluid. Trained microbiology technicians analyzed BAL fluid by smear and culture for mycobacteria, *Pneumocystis*, and other fungi according to standard protocols. Full details of bronchoscopy and specimen examination are described in the Online Supplement.

At discharge, we asked patients to return in two months for a follow-up clinical examination and repeat sputum analysis. After participants had completed all study procedures and the two-month follow-up visit, at least two pulmonary physicians (AC, JLD, LH, WW, and SY) reviewed all available clinical and microbiologic data, and assigned final diagnoses according to explicit clinical definitions (Online Supplement S1).

### Statistical Analysis

We calculated risk ratios, sensitivities, specificities, and positive and negative likelihood ratios to measure the performance of clinical and radiographic variables for diagnosing TB, in reference to a gold standard of any positive sputum or BAL fluid culture. Although the sample size arose from convenience, we determined the precision of all diagnostic accuracy estimates using exact binomial confidence intervals in lieu of power calculations. [9] To contrast the clinical utilities of clinical and radiographic factors for either diagnosing or excluding TB, we categorized characteristics as either predominantly “sensitive” or predominantly “specific.” We subjectively defined characteristics whose sensitivity estimates were higher than their specificity estimates as “sensitive” tests, and characteristics whose specificity estimates were higher than their sensitivity estimates as “specific” tests.

To evaluate whether a combination of characteristics would be highly predictive of TB, we constructed a multivariate model including only the 14 factors with the greatest face validity in order to optimize the balance between bias and variance. [10] We used conventional logistic regression to generate predicted probabilities of TB, plotted the fraction of true positives versus the fraction of false positives at various thresholds of predicted probability as a receiver operating characteristic curve, and measured model accuracy using the concordance (C) statistic (area under the curve). We adjusted our estimate of the C statistic for optimism using ten-fold cross-validation with random re-sampling to generate average predicted probabilities. [11] We investigated model misspecification by examining the significance of added quadratic terms using the Wald test, and we assessed model calibration using the Hosmer-Lemeshow test. We performed several sensitivity analyses. We assessed the impact of excluding patients who did not undergo chest radiography by building a model based on clinical variables only. Similarly, we re-ran our multivariate model including those with unknown culture status, once assuming these individuals had TB, and a second time assuming that they did not have TB. Last, to test the vulnerability of our models to outcome misclassification, we performed a sensitivity analysis in which we classified patients with clinically defined culture-negative TB as having culture-positive TB, and another in which we excluded these patients entirely.

Finally, we built one additional multivariate model using the Deletion/Substitution/Addition (DSA) algorithm [12], a novel data-adaptive estimation routine that considers non-linear terms and all possible interactions between predictors while simultaneously avoiding overfitting through repeated cross-validation. We used STATA version 10.0 (Stata Corporation, College Station, Texas) and R version 2.8.1 (R Project for Statistical Computing) for our calculations.

## Results

### Study Population

**Figure 1:**
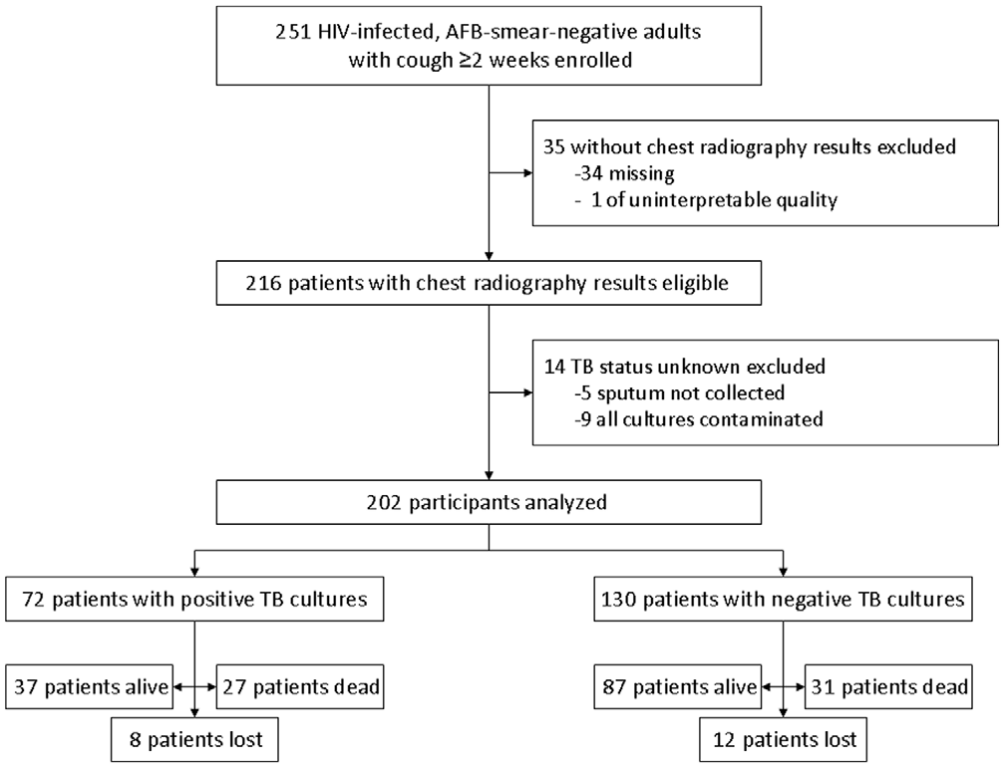
Patient enrollment and outcome flow diagram. The diagram presents the total numbers of patients enrolled, eligible, and analyzed, and the vital status of all patients at two-month follow-up.

Of 407 patients enrolled, 251 (62%) were eligible for this analysis by having negative sputum smears for AFB (Figure 1). The other 156 patients had positive AFB smears, and 147 (94%) had clinically and/or bacteriologically confirmed TB. Of the 251 smear-negative patients, 216 (86%) had chest radiography results. Of the 35 sputum smear-negative patients missing chest radiography results, one had an uninterpretable chest radiograph, and 34 never underwent chest radiography, most commonly because the radiology department was too busy at the time of admission or because the patient was too weak to travel there from the emergency ward. We excluded 14 additional subjects because mycobacterial culture results were indeterminate (nine had both sputum cultures contaminated and five could not produce a respiratory specimen), leaving an eligible population of 202 HIV-infected, sputum smear-negative patients with chest radiography results. In addition, four patients with unknown CD4+ T-cell counts were excluded from the DSA analysis.

As shown in Table 1, patients were generally young, with a median age of 32 years (inter-quartile range (IQR) 28–39). Most had advanced AIDS, with a median CD4+ T-cell count of 64 cells/µL (IQR 23–191). Although a high proportion (74%, 149 patients) had known of their HIV infection prior to admission, only 18% (36 patients) were taking antiretroviral therapy. One-hundred thirty-four (66%) patients had been treated with antibiotics prior to admission without response. One-hundred nineteen (59%) patients underwent bronchoscopy; 83 (41%) patients did not undergo bronchoscopy for the following reasons: an alternate diagnosis was independently confirmed or the patient improved on empiric therapy and was discharged (38, 19%); the patient refused (23, 11%); or the patient was too ill or died before the procedure (22, 11%).

**Table 1:** Demographic and clinical characteristics of the 202 study participants.

Characteristic	All Patients	TB*	No TB	P-value
	(n = 202)	(n = 72)	(n = 130)	
Median age (IQR), years	32 (28–39)	32 (28–39)	33 (28–40)	0.59
Female sex (%)	113 (56)	38 (53)	75 (58)	0.50
Newly diagnosed with HIV (%)	53 (26)	14 (19)	39 (30)	0.10
Median CD4 count (IQR), cells/µL†	64 (23–191)	60 (17–148)	74 (26–213)	0.17
Taking co-trimoxazole prophylaxis (%)¶	117 (58)	48 (67)	69 (53)	0.061
Taking antiretroviral therapy (%)§	36 (18)	15 (21)	21 (16)	0.41
Took antibiotics prior to admission (%)	134 (66)	51 (71)	83 (64)	0.31
Two-month mortality (%)‡	58 (32)	27 (42)	31 (26)	0.028

**Abbreviations:** AFB, acid-fast bacilli; IQR, inter-quartile range; %, percent; TB, tuberculosis; µL, microliter.

**Legend:** *TB defined by any positive sputum or bronchoalveolar lavage mycobacterial culture on solid media.

† 4 responses missing.

¶ All but one patient had been taking co-trimoxazole for ≥1 month.

§ All patients reported taking antiretroviral therapy for ≥1 month.

‡ 8 patients with TB and 12 patients without TB were lost to follow-up.

### Diagnoses and Outcomes

In 72 (36%) patients, sputum and/or BAL cultures were positive for TB, and in 130 (64%) patients, they were negative. Sixty-one (30%) patients were positive on both sputum and BAL fluid cultures, and eleven (6%) were positive on BAL fluid culture alone. Although their cultures were negative, 14% (29 patients, 18 with negative sputum and BAL fluid cultures and 11 with negative sputum cultures only) were classified as having culture-negative TB, either because they improved with TB treatment and had negative sputum cultures at the two-month follow-up visit (16 patients, 8%), or because their two-month follow-up cultures were positive (13 patients, 6%). Of the remaining 101 (50%) patients without a clinical or microbiologic diagnosis of pulmonary TB, 59 (29%) patients had one or more of the following diagnoses: 41 (20%) had bacterial pneumonia, 8 (4%) had cryptococcal pneumonia, 5 (2.5%) had Kaposi's sarcoma, 4 (2%) had *Pneumocystis* pneumonia, and 3 (1.5%) had other non-infectious or extra-pulmonary explanations for their symptoms. More than one diagnosis was present in two of these 59 patients (1%), and the final diagnosis was unknown in the remaining 42 (21%) patients. Fifty-eight patients (32%) died within two months of hospital admission, and 20 (10%) were lost to follow-up. Those with culture-proven TB had a significantly higher mortality at two months than non-TB patients (42% vs. 26%, Risk Ratio 1.6, 95% Confidence Interval (CI) 1.1–2.4, p = 0.028).

### Individual Factors with a High Sensitivity or Specificity for Tuberculosis

No individual clinical or radiographic factor was both sensitive and specific for TB. Among individual symptoms, subjective fever, weight loss, sputum production, and absence of hemoptysis were more sensitive for TB than specific (Table 2). A CD4+ T-cell count <200 cells/µL and chest radiography interpreted by a radiologist as consistent with TB were also more sensitive for TB than specific. Among all of these factors, weight loss (96%) and fever (93%) had the highest sensitivities for TB.

**Table 2:** Diagnostic performance of clinical and radiographic characteristics with high sensitivity for tuberculosis.

Characteristic	Frequency	TB*	No TB	Risk Ratio	Sensitivity	Specificity	LR (+)	LR (−)
(n = 202)	(%)	(n = 72)	(n = 130)	(95% CI)	(95% CI)	(95% CI)	(95% CI)	(95% CI)
History of fever	192 (95)	67	125	0.70	93	3.9	0.97	1.8
				(0.36–1.3)	(85–98)	(1.3–8.8)	(0.90–1.0)	(0.54–6.0)
Weight loss	185 (92)	69	116	2.1	96	11	1.1	0.39
				(0.74–6.0)	(88–99)	(6.0–17)	(1.0–1.2)	(0.12–1.3)
Sputum production	171 (85)	60	111	0.91	83	15	0.98	1.1
				(0.56–1.5)	(73–91)	(9.0–22)	(0.86–1.1)	(0.59–2.2)
Absence of hemoptysis	143 (71)	58	85	1.7	81	35	1.2	0.56
				(1.0–2.8)	(70–89)	(27–44)	(1.0–1.5)	(0.33–0.95)
Dyspnea	118 (58)	37	81	0.75	51	38	0.83	1.3
				(0.52–1.1)	(39–63)	(29–47)	(0.64–1.1)	(0.93–1.8)
Abnormal breath sound	143 (71)	49	94	0.88	68	28	0.94	1.2
				(0.59–1.3)	(56–79)	(20–36)	(0.78–1.1)	(0.75–1.8)
CD4 count <200 cells/µL†	151 (76)	57	94	1.4	81	27	1.1	0.70
				(0.82–2.3)	(70–90)	(19–35)	(0.95–1.3)	(0.40–1.2)
Radiologist's diagnosis of TB	157 (78)	56	101	1.0	78	22	1.0	1.0
				(0.64–1.6)	(66–87)	(16–30)	(0.86–1.2)	(0.58–1.7)

**Abbreviations:** 95% CI, 95% Confidence Interval; LR(+), positive likelihood ratio; LR (−), negative likelihood ratio; SD, standard deviation; TB, tuberculosis; %, percent; µL, microliter.

**Legend:** *TB defined by any positive sputum or bronchoalveolar lavage mycobacterial culture on solid media.

† 4 responses missing.

In contrast, objective fever (body temperature ≥38.3°C) at the time of evaluation was more specific than sensitive for TB (Table 3). The radiographic presence of pleural effusion, cavities, and hilar adenopathy were also more specific than sensitive for TB. Among all of these factors, the presence of pleural effusion (98%) and cavities (97%) had the highest specificities for TB.

**Table 3:** Diagnostic performance of clinical and radiographic characteristics with high specificity for tuberculosis.

Characteristic	Frequency	TB*	No TB	Risk Ratio	Sensitivity	Specificity	LR (+)	LR (−)
(n = 202)	(%)	(n = 72)	(n = 130)	(95% CI)	(95% CI)	(95% CI)	(95% CI)	(95% CI)
Temperature ≥38.3°C	26 (13)	15	11	1.8	21	92	2.5	0.87
				(1.2–2.6)	(12–32)	(85–96)	(1.2–5.1)	(0.76–0.98)
Respiratory rate ≥30	62 (31)	20	42	0.87	28	68	0.86	1.1
				(0.57–1.3)	(18–40)	(59–76)	(0.55–1.4)	(0.89–1.3)
Oxygen saturation <93%	53 (26)	19	34	1.0	26	74	1.0	1.0
				(0.66–1.5)	(17–38)	(65–81)	(0.62–1.6)	(0.84–1.2)
Hilar adenopathy	31 (15)	10	21	0.89	14	84	0.86	1.0
				(0.51–1.5)	(6.9–24)	(76–90)	(0.43–1.7)	(0.91–1.2)
Pleural effusion	26 (13)	12	14	1.4	17	89	1.6	0.93
				(0.85–2.2)	(8.9–27)	(83–94)	(0.76–3.2)	(0.83–1.1)
Cavity	8 (4.0)	3	5	1.1	4.2	96	1.1	1.0
				(0.42–2.6)	(0.87–12)	(91–99)	(0.27–4.4)	(0.94–1.1)

**Abbreviations:** 95% CI, 95% Confidence Interval; LR(+), positive likelihood ratio; LR (−), negative likelihood ratio; TB, tuberculosis; %, percent.

**Legend:** *TB defined by any positive sputum or bronchoalveolar lavage mycobacterial culture on solid media.

Other clinical factors, including dyspnea, hypoxemia, tachypnea, and abnormal breath sounds were neither sensitive nor specific for TB. Except for objective fever, present in only 26 (13%) patients, and absence of hemoptysis in 143 (71%) patients, no clinical or radiographic factor achieved a positive or negative likelihood ratio statistically different from 1.0, a value which provides no diagnostic information. [16] Even after stratifying patients by CD4+ T-cell count (≥200 or <200 cells/ul), there were no characteristics which provided information that was clinically useful for diagnosing or excluding TB.

### Clinician Judgment

Forty-five (25%) of the 177 patients who survived to hospital discharge were empirically started on standard TB treatment after case review by National TB and Leprosy Control Programme clinicians; sputum or BAL cultures turned positive for 23 (51%). Of the 132 patients not started on treatment, sputum or BAL cultures turned positive for 40 (30%). The sensitivity of a clinician decision to initiate TB treatment prior to hospital discharge was 37% (95% CI 25–50%) and specificity was 81% (95% CI 73–88%), with a positive likelihood ratio of 1.9 (95% CI 1.2–3.1) and a negative likelihood ratio of 0.79 (95% CI 0.64–0.97).

### Multivariate Prediction Models

**Figure 2:**
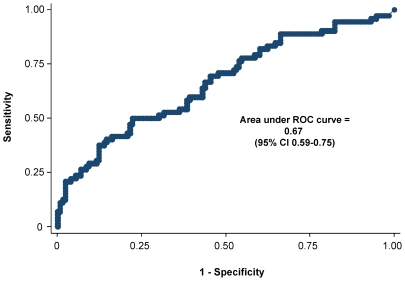
Receiver operating characteristic (ROC) curve. The sensitivity versus one minus the specificity of the cross-validated multivariable logistic regression model for diagnosing tuberculosis developed in this study is plotted at every threshold of probability of tuberculosis predicted by the model.

We developed a multivariable prediction model by regressing all of the aforementioned clinical and radiographic variables on the log odds of a positive mycobacterial culture. The model had acceptable calibration (p = 0.55 by the Hosmer-Lemeshow test) and was not sensitive to outliers or model misspecification (p = 0.40 by the Wald test). As with the analyses of diagnostic accuracy for single variables, a multivariate model including all 14 predictors found in Tables 2 and 3 failed to identify any large or statistically significant associations with culture-positive TB. A receiver operating characteristic curve for this combination of predictors (Figure 2) confirmed its poor clinical performance (C statistic 0.67, 95% CI 0.59–0.75). Sensitivity analyses incorporating patients with missing radiographic or culture results into the model did not change the results across a full range of assumptions. Similarly, neither reclassifying culture-negative TB patients as having culture-positive TB nor excluding them from the analysis changed any of these findings.

To test further the possibility that certain combinations of variables from the full set of measured clinical and radiographic predictors might be clinically useful for diagnosing TB, we generated another prediction model using the Deletion/Substitution/Addition (DSA) routine. In examining 30 different clinical and radiographic variables, no combination, with or without interaction terms, outperformed a model including only the model intercept. In other words, no clinical prediction rule could be identified that was more accurate than randomly assigning diagnoses of TB in proportion to the TB prevalence in the study population.

## Discussion

Employing both conventional logistic regression and a novel data-adaptive estimation routine, we failed to identify any clinical or radiographic factor or combinations of factors with useful performance characteristics for diagnosing TB among hospitalized, HIV-infected, smear-negative adults suspected of TB in a low-income country with a high incidence of TB. Even a clinician decision to initiate TB treatment at hospital discharge performed only marginally better than chance. Among the 202 patients who were fully evaluated for TB in accordance with World Health Organization (WHO) guidelines for the assessment of smear-negative patients in HIV-prevalent settings [2], one-third died within two months of hospital admission. These findings emphasize the need for studies of faster and more sensitive diagnostic strategies for evaluating smear-negative patients, including trials that measure the success of new diagnostics against that of empiric treatment for TB in individuals at high risk of death. [4]

The usefulness of a diagnostic test will depend on a number of factors, including the prevalence (or incidence) of the disease in the population sample, the frequencies with which the test is positive in patients with the disease (sensitivity) and negative in patients without the disease (specificity), and the physical risk and financial expense associated with the test and any resulting treatment. Within this framework, symptoms and physical findings are no different from any other diagnostic test: characteristics subjectively defined as sensitive are useful in excluding disease, especially when the disease is uncommon in the population tested; and characteristics subjectively defined as specific are useful in diagnosing disease, especially when the disease is common in the population tested. [17] Given the comparatively low cost and low risks of using symptoms, physical findings, and even chest radiography to diagnose TB, these tests hold natural appeal to clinicians practicing in a high TB-incidence, low-income country like Uganda—particularly since no other tests are routinely available. Combining the information given by sensitivity and specificity, positive likelihood ratios >2 and negative likelihood ratios <0.5 represent the minimal diagnostic performance needed to increase or decrease the post-test probability of a given diagnosis by 15%, the minimal change in probability likely to be clinically important. [16] By these standards, our data suggest that, except for the rare finding of objective fever (present in only 13%), the clinical characteristics we evaluated are neither sensitive nor specific enough to change the likelihood of TB in an individual patient.

There are a number of possible explanations why, in spite of evidence in the literature from sub-Saharan Africa and other settings [18], [19], [20], physical signs, symptoms, and radiographic findings did not predict TB in our setting. First, although previous studies included some HIV-infected patients [21], our study included *only* HIV-infected patients, most of whom had advanced AIDS. These patients are at high risk of other opportunistic pulmonary conditions which may present in isolation or in combination with bacterial and mycobacterial infections. The similarity of symptoms, signs, and radiographic findings across this etiological spectrum, especially when multiple conditions are present, may reduce the specificity of classical findings for the diagnosis of TB. Second, the high value of clinical and radiographic factors for screening high-risk populations for TB (“active case finding”) must be distinguished from their low value in diagnosing symptomatic patients suspected of TB (“passive case finding”). [22] For TB screening among HIV-infected populations initiating antiretroviral therapy, for example, much evidence supports the usefulness of clinical and/or radiographic characteristics. [5], [7], [23], [24], [25] For evaluating symptomatic patients suspected of TB, clinical characteristics may be useful for triage of coughing patients to more sensitive (chest radiography) [26] or more specific (sputum smear microscopy; antibiotic trials) tests [27], [28], [29], but investigators have been unable to find algorithms that are both highly sensitive and highly specific for confirming TB, either because proposed models had poor reproducibility in cross-validation samples [30], or because model complexity reduced ease of use. [31] Third, several studies have used non-standard measures of diagnostic performance such as odds ratios to report their findings [19], [32], [33], [34], [35], rather than appropriate measures such as sensitivity, specificity, and likelihood ratios. Odds ratios overestimate the performance of clinical and radiographic factors because they do not differentiate between false-positive and false-negative test results. In addition, even odds ratios that are large in epidemiologic terms may not indicate high levels of diagnostic accuracy. [36]

There were several strengths to the study. First, the study enrolled consecutive HIV-infected patients suspected of TB who had been found to have negative sputum AFB smears while undergoing routine evaluation. Because sputum smear microscopy fails to detect almost half of all TB cases, patients with negative smears represent a highly relevant population for whom a number of clinical guidelines have been developed. [2], [3] Second, since clinicians with routine experience caring for patients in the study hospital performed all measurements, the accuracy of the clinical and radiographic measurements likely reflects or exceeds that found in everyday practice. Third, we used a variety of rigorous and exhaustive model construction methods to search for any possible clinical or radiographic algorithm that might be predictive. This approach makes it unlikely that our study could be falsely negative. Although other investigators have used similar techniques to identify clinically accurate algorithms [25], [31], [37], our use of advanced prediction methods with cross-validation applies a relatively novel approach to diagnosing TB in symptomatic patients in a low-income country with a high prevalence of HIV, where the demand for such algorithms is the greatest. Finally, we performed multiple sensitivity analyses to be sure that misclassification of TB status did not affect the internal validity of our findings.

There were several limitations to the study. First, we studied a severely ill population of inpatients, most of whom had failed a course of antibiotics prior to admission. Although our findings may thus appear less relevant than those from outpatient settings, our patients are likely representative of the referral population envisaged in two relevant WHO guidelines. These recommend that smear-negative patients suspected of TB who fail to improve after empiric antibiotic treatment be referred to a “hospital [for] further assessment by a more senior clinician” [3], with the decision to treat for TB to be based on “clinical assessment.” [2] Although our exclusion of some patients who were missing chest x-rays–perhaps because they were more severely ill–may also be considered a limitation, the proportion was small (14%), and a secondary analysis looking at clinical factors alone with inclusion of these patients also failed to identify any highly predictive factors for diagnosing TB.

Second, although our gold standard rigorously included multiple sputum and/or BAL fluid cultures, we may have misclassified some culture-negative patients as not having TB. However, our study also included two-month follow-up of all patients, and we were able to determine that the overall proportion (15%) of patients with culture-negative TB in our study was small. Moreover, neither reclassifying nor leaving these patients out of the analysis changed our conclusions. Third, our sample size may have been inadequate to generate an adequate prediction model. However, the precision estimates for all diagnostic performance measures (accuracy, likelihood ratios, and the area under the receiver operating characteristic curve) show that even a larger sample is unlikely to identify variables which are clinically informative for diagnosing TB. Finally, it may be impossible to divide the diagnostic value of a clinical impression into discrete variables. Highly experienced and skilled clinicians may be able to incorporate clinical and radiographic factors to diagnose TB accurately in a manner that cannot be captured in our analyses. However, we found that clinicians routinely practicing in this setting were also unable to predict TB, as judged by the low predictive value of their decisions to initiate TB treatment in this study. Thus, while clinical and radiographic algorithms may be appropriate for screening patients for TB in settings with access to specific confirmatory tests such as culture, the results of this and other operational studies argue against their use for routine diagnosis of symptomatic, HIV-infected, smear-negative patients suspected of TB. Instead, researchers and policy makers should focus on enhancing the speed and sensitivity of smear microscopy through streamlined sputum collection strategies [38], [39], light-emitting diode fluorescence imaging [40], [41], and improved quality assurance. [42], [43]

In conclusion, clinicians evaluating hospitalized HIV-infected, AFB smear-negative TB suspects face a tremendous challenge in determining the causes of respiratory complaints in these patients. For patients who lack danger signs, including tachypnea, tachycardia, fever, and inability to walk, guidelines recommend expert opinion in determining whether to initiate TB treatment. [2] Our findings suggest that discrete clinical or radiographic criteria are poorly predictive of culture-proven TB in a population in which advanced AIDS is highly prevalent. In addition, the exceptionally high early mortality among TB patients (42% at two months) emphasizes the urgent need for diagnostic methods for smear-negative TB which are more accurate than clinical criteria yet equally rapid and affordable in low-income settings with a high incidence of HIV and TB.

## Supporting Information

Online Supplement S1 Supplementary details on research methods, diagnosis assignment algorithms, and the chest radiograph interpretation form are provided to the reader.
(0.19 MB DOC)
