# Supplementary material for: Clinical and Radiographic Factors Do Not Accurately Diagnose Smear-Negative Tuberculosis in HIV-infected Inpatients in Uganda: A Cross-Sectional Study
Source: PLoS One. 2010 Mar 26;5(3):e9859. doi: 10.1371/journal.pone.0009859 (PMC2845634; doi:10.1371/journal.pone.0009859)
Supplement: Online Supplement S1 — Supplementary details on research methods, diagnosis assignment algorithms, and the chest radiograph interpretation form are provided to the reader. (0.19 MB DOC) [file pone.0009859.s001.doc]

**Specimen Collection**

Each participant provided blood for determination of the CD4+ T-lymphocyte count, and expectorated sputum on enrollment (“spot”) and on the following morning (“early morning”). A research assistant delivered sputum samples to the Uganda National TB Reference Laboratory (NTRL), where experienced technicians examined each specimen by direct light microscopy and concentrated fluorescence microscopy according to standard protocols.[1] Processed sputum specimens were inoculated on two Lowenstein-Jensen slants. Cultures were read weekly and considered positive if any growth (≥ 1 colony forming unit) was identified within eight weeks.

Patients with negative direct Ziehl-Neelsen or concentrated fluorescence smears (less than 10 organisms per 100 fields)[2,3] were referred for bronchoscopy with airway inspection (to identify Kaposi’s sarcoma lesions) and BAL. The decision to perform bronchoscopy was left to the treating chest physicians, and required a separate clinical consent process.

**Bronchoscopy**

Clinical investigators (WW, JLD, AC, SY) performed all bronchoscopies according to a standard protocol, which included monitoring with continuous pulse oximetry and provision of supplemental oxygen as needed. After administering nebulized and topical 1% lidocaine for airway anesthesia, and intramuscular midazolam for anxiolysis, bronchoscopists performed BAL by wedging the tip of the bronchoscope into a subsegment of lung with the greatest radiographic abnormality. Sterile, non-bacteriostatic normal saline (0.9%) was instilled in serial 25 mL aliquots (up to a maximum of 125 mL) and then aspirated until at least 50 mL were returned. BAL fluid was immediately divided and sent for microbiologic testing. Bronchoscopists also inspected the trachea and all mainstem and segmental bronchi for Kaposi’s sarcoma lesions, which we diagnosed if we identified one or more focal or confluent macules, nodules, patches, or plaques with shiny black, purple, or blue hyperpigmentation.

**Specimen Analysis**

A trained laboratory technologist (AA) examined a concentrated pellet from a minimum of 10 mL of BAL fluid for *Pneumocystis jirovecii* using a modified Giemsa stain (Diff-Quik, Dade-Behring, Newark, DE). At least 2 mL of BAL fluid was delivered to the Mulago Hospital Microbiology Laboratory for testing for other fungi using a potassium hydroxide stain (KOH) and culture on Sabouraud’s agar. The NTRL examined a minimum of 5 mL of BAL fluid for mycobacteria using concentrated fluorescence microscopy and Lowenstein-Jensen culture. The study team communicated all bronchoscopic and microbiologic results to patients and/or their treating clinicians as soon as results became available.

**Quality Assurance**

All sputum and BAL smears were read by one of five full-time laboratory technicians who have a median experience of 10 years (range 5-20 years). Since 2005, the Uganda National TB Lab has participated in a biannual external quality assurance (EQA) program for smear microscopy administered by the WHO. The EQA program consists of a panel of 5 stained and 5 unstained conventional light microscopy slides of various grades, and the National TB Lab has passed all quality assurance rounds. Known positive and negative control slides were included with each batch of slides read. Only sterile, distilled water was used to prepare all reagents. A sample of Giemsa slides were re-read blindly in the Department of Microbiology at San Francisco General Hospital.

**Protocol for Assigning Final Diagnoses**

Final diagnoses were assigned by review of the following information:

1. Results of spot and early morning sputum mycobacterial cultures at time of enrollment;
2. Results of chest radiography;
3. Results of bronchoscopy inspection, stains and non-mycobacterial cultures;
4. Results of empiric anti-tuberculosis (TB) treatment or antibiotic treatment trials (whether the patient improved from baseline to the two-month visit, in the patient’s or clinician’s judgment);
5. Results of sputum smears and cultures at the two-month follow-up visit.

Possible diagnoses included:

1. Pulmonary TB (culture-positive and culture-negative)
2. Extra-pulmonary TB
3. Pulmonary Kaposi’s sarcoma (KS)
4. *Pneumocystis jirovecii* pneumonia (PCP)
5. Pulmonary cryptococcosis (Crypto PNA)
6. Pulmonary aspergillosis (Aspergillus PNA)
7. Bacterial pneumonia (BPNA)
8. Acute bronchitis
9. Other
10. Unknown.

All diagnoses were assigned in a hierarchical fashion, with TB status assigned first, and all other diagnoses assigned second according to the algorithm shown below. Cases with an uncertain diagnosis and/or atypical clinical circumstances were presented to the research team and at least two pulmonary physicians (AC, JLD, LH, WW, SY), and a final diagnosis assigned by consensus.

**(1) Pulmonary TB (PTB)**

- **Culture-positive TB** was defined as having a positive sputum or BAL culture growing one or more colony forming units of bacilli morphologically consistent with *M.TB*.
- **Culture-negative TB** was defined in one of two ways. Patients who improved from baseline to two months with continuous treatment with anti-TB drugs with all mycobacterial cultures being negative. In addition, patients with negative cultures whose cough and respiratory symptoms failed to improve from baseline to two months but whose 2-month sputum culture grew one or more colony forming units of bacilli morphologically consistent with *M.TB* were also classified as having culture-negative TB.
- **Non-TB status** was defined as having negative baseline cultures for *M.TB* and improvement without TB treatment with follow-up smears and cultures not positive for *M.TB*; as having negative baseline cultures for *M.TB* without improvement without TB treatment and negative follow-up smears and follow-up cultures not positive for *M.TB*; or, as having negative baseline cultures for *M.TB* without improvement with TB treatment and follow-up smears negative and follow-up cultures not positive for *M.TB*.
- **Indeterminate TB status** was defined as having negative cultures for *M.TB* and dying or being lost to follow-up prior to the two month visit; as having no improvement with or without treatment for TB and a non-negative smear and an unknown follow-up culture result for *M.TB*; or as having no improvement with treatment but a positive two-month sputum smear and a negative two-month sputum culture for *M.TB*. In addition, those with unknown baseline culture status, no or unknown improvement with TB treatment, and an unknown two-month sputum culture result were described as having indeterminate TB status.

**Table S1.** Definitions of TB Status Categories.

| **TB Category** | **Baseline Sputum/BAL Culture** | **Treated for TB?** | **Improved at 2 months?** | **2-month Sputum Smear** | **2-month Sputum Culture** |
| --- | --- | --- | --- | --- | --- |
| **Culture-positive TB** | >1 CFU | --* | -- | -- | -- |
| **Culture-negative TB** | <1 CFU | Yes | Yes | -- | -- |
|  | <1 CFU | -- | -- | -- | >1 CFU |
| **Non-TB Status** | Negative | No | Yes | Negative, or Not collected | Negative, or Contaminated |
|  | Negative | No | No | Negative | Negative, or Contaminated |
|  | Negative | Yes | No | Negative | Negative, or Contaminated |
| **Unknown TB Status** | Negative, or Contaminated | Unknown | -- | -- | -- |
|  | Negative, or Contaminated | -- | Died, or Lost | -- | -- |
|  | Negative, or Contaminated | No | -- | Positive, or Not collected | Contaminated, or Not collected |
|  | Negative, or Contaminated | Yes | No | Positive, or Not collected | Negative, Contaminated, or Not collected |
|  | Missing | -- | No, or Died, or Lost | -- | Contaminated, or Not collected |

**Legend:** *-- denotes that the result contained within does not affect the classification scheme.

**2) Extra-pulmonary TB (EPTB)**

- Clinical evidence of extra-pulmonary TB by one of the following, AND
  - TB meningitis by cerebrospinal fluid chemistries;
  - TB adenitis by lymphadenopathy on physical exam, ultrasound or histopathology;
  - TB pericarditis by chest radiography or echocardiography;
  - Miliary TB by chest radiography
  - TB pleuritis by pleural aspirate chemistries;
  - TB ascites by peritoneal aspirate chemistries;
  - Spinal TB by plain radiography
- Clinical response to treatment at 2 months.

**(3) *Pneumocystis* pneumonia (PCP)**

- Positive Giemsa stain on BAL or induced sputum

**(4) Cryptococcal Pneumonia (CryptoPNA)**

- Positive BAL KOH stain or positive culture on Sabouraud’s agar for *Cryptococcus*

**(5) Pulmonary Kaposi’s sarcoma (PKS)**

- Typical bronchial lesions on bronchoscopy

**(6) *Aspergillus* Pneumonia**

- Positive BAL culture for Aspergillus

**(7) Bacterial pneumonia (BPNA), or Acute bronchitis**

- Clinical criteria
  - No alternate diagnosis, AND
  - Clinical improvement at 1-2 weeks or 2 months , AND
  - All acid-fast bacilli cultures negative
- Radiographic criteria
  - Pulmonary infiltrates on chest radiography?
    - Yes: bacterial pneumonia
    - No: acute bronchitis

**(8) Other**

- This diagnosis is assigned if the patient has an infectious illness not mentioned above or any non-infectious illness that explains all of their pulmonary symptoms

**(9) Unknown**

- This diagnosis is assigned if the patient fails to meet any of the above criteria.

**REFERENCES FOR ONLINE REPOSITORY**

S1. Kent PT, Kubica GP (1985) Public Health Mycobacteriology. A Guide for the Level III Laboratory. Atlanta: Centers for Disease Control.

S2. (1998) Laboratory Services in Tuberculosis Control: Microscopy Part I. Geneva, Switzerland: World Health Organization. WHO/TB/98.258. WHO/TB/98.258. .

S3. Rieder HL, Van Deun A, Kam KM, Kim SJ, Chonde TM, et al. (2007) Priorities for Tuberculosis Bacteriology Services in Low-Income Countries. Paris: International Union Against Tuberculosis and Lung Disease.

**FORM 9 - MIND STUDY**

“.” if N/A

0a. **←**

0b. ⁭

1. ⁭

**CHEST RADIOGRAPH INTERPRETATION DATA**

1. a. Date______/______/_______

b. What is the overall quality of the film?

HIGH……………………………. 0 POOR………………………. 2

ACCEPTABLE…………………. 1 UNREADABLE……………. 3

1. Does the parenchyma appear abnormal?

NO …………….……………….............…………..................... 0 (Go to #12)

YES ………….………………..……………………………….. 1 (Go to #2)

2-7. Please describe the infiltrate in each of the following zones using the following codes:

|  | **a. Pattern**  Normal 0  Mass 1  Reticulolinear 2  Reticulonodular 3  Nodules 4  Ring opacity 5  Ground Glass 6  Consolidation 7  Hyperlucent 8  Linear band 9  Pleural opacity 10  Mixed (specify b) 11  Other (specify c) 12 | **Fill only if the answer for “a. Pattern” is…** | |
| --- | --- | --- | --- |
|  |  |  |  |
|  |  | **b. Mixed**  (List patterns using + sign; e.g. 2+3+4) | **c. Other** |
| 2. RU Lung Zone | a. | b. | c._________________________________ |
| 3. RM Lung Zone | a. | b. | c.____________________________________ |
| 4. RL Lung Zone | a. | b. | c.____________________________________ |
| 5. LU Lung Zone | a. | b. | c.____________________________________ |
| 6. LM Lung Zone | a. | b. | c.____________________________________ |
| 7. LL Lung Zone | a. | b. | c.____________________________________ |

1. Consolidation

“.” if N/A

1. ⁭

9a. ⁭

9b. ⁭

9c. ⁭

10a. ⁭

10b. ⁭

10c. ⁭

10d. ⁭

10e ⁭

11a. ⁭

11b. ⁭

11c. ⁭

11d. ⁭

11e. ⁭

HOMOGENOUS……………………………………… 0

PATCHY………………………………………………. 1

1. Nodule Details (Specify only if answer to questions 2-7 includes “4”)
   1. Number

SOLITARY……………………………………………. 0

MULTIPLE……………………………………………. 1

- 1. Size

MILIARY (<2mm)…………………………………… 0

SMALL (2-3mm)………………………………………. 1

MEDIUM (>3-5mm)…………………………………….. 2

LARGE (>5mm)………………………………………... 3

- 1. Calcification

NON-CALCIFIED……………………………………… 0

CALCIFIED…………………………………………….. 1

BOTH…………………………………………………… 2

1. Cavity Details (Specify only if answer to questions 2-7 includes “5”)
   1. Number

SOLITARY……………………………………………. 0

MULTIPLE……………………………………………. 1

- 1. Size

SMALL (< 2cm)………………………………………. 1

MEDIUM (2-5cm)……………………………………. 2

LARGE (>5cm)……………………………………….. 3

- 1. Wall Thickness

THIN (≤ 1mm)………………………………………… 0

MODERATE (1-3mm)………………………………… 1

MARKED (>3mm)……………………………………. 2

- 1. Fluid Levels

ABSENT……………………………………………….. 0

PRESENT………………………………………………. 1

- 1. Intracavitary bodies

ABSENT………………………………………………. 0

PRESENT………………………………………………. 1

1. Predominant location of the findings on the radiograph as a whole?
   1. Distribution

FOCAL DISEASE……………………………………….… 0

DIFFUSE DISEASE ………………………………………. 1

- 1. If FOCAL DISEASE

UPPER LOBE…………………………………………... 0

MIDDLE LOBE………………………………………… 1

LOWER ………………………………………............... 2

- 1. CENTRALITY OF DIFFUSE

CENTRAL…………………………………………… 0

PERIPHERAL………………………………….. ….. 1

UNIFORM……………………………………… ….. 2

- 1. Symmetry

BOTH…………………………………………………… 0

RIGHT……....………………………………………….. 1

LEFT………………....…………………………………. 2

- 1. Simplified distribution

DIFFUSES………………………………………………. 0

UPPER LOBE……………………………………………. 1

LOWER LOBE…………………………………………… 2

1. Cardiac silhouette

“.” if N/A

12. ⁭

13. ⁭

14. ⁭

15. ⁭

16. ⁭

16a. ⁭

17. ⁭

17b. ⁭

17c. ⁭

17d. ⁭

17e. ⁭

17f. ⁭

17g. ⁭

20. ⁭

20a. **←**

21. ⁭

NORMAL………………………………………………. 0

ENLARGED……………………………………………. 1

UNINTERPRETABLE……………………………… 2

1. Mediastinal Position

MIDLINE………………………………………………. 0

SHIFTED RIGHT……………………………………… 1 SHIFTED LEFT………………………………………… 2

1. Mediastinal size

NORMAL………………………………………………. 0

WIDENED…………………………………………….. 1

MASS………………………………………………….. 2

1. Hilar Elevation

NONE…………………………………………………... 0

RIGHT………………………………………………….. 1

LEFT……………………………………………………. 2

BOTH…………………………………………………… 3

1. Is Hilar/mediastinal lymphadenopathy present?

NO …………….……………….............…………......... 0 (Go to #17)

YES ………….………………..……………………….. 1 (Go to #16a)

- 1. Location?

BOTH………………………………………………….. 0

RIGHT……....…………………………………………. 1

LEFT………………....………………………………… 2

1. PLEURAL ABNORMALITIES
   1. Apical cap?

NO …………………….............…………...................... 0

YES ….………………..……………………………….. 1

- 1. Pleural thickening

ABSENT………………………………………………. 0

RIGHT……....…………………………………………. 1

LEFT………………....………………………………… 2

BOTH………………………………………………….. 3

- 1. Effusion

ABSENT………………………………………………. 0 (Go to 17f)

RIGHT……....…………………………………………. 1 (Go to 17d)

LEFT………………....………………………………… 2 (Go to 17d)

BOTH………………………………………………….. 3 (Go to 17d)

- 1. Effusion Size

SMALL (Less than one lung zone)………..................... 0

MODERATE (btw 1 and 2 lung zones).......................... 1

MASSIVE (> 2 lung zones).………............................... 2

- 1. Effusion Type

FREE…………………………………………………… 0

LOCULATED…………………………………………. 1

- 1. Pneumothorax

ABSENT…………………………………………… 0 (Go to 18)

RIGHT……………………………………………… 1 (Go to 17g)

LEFT ………………………………………………. 2 (Go to 17g)

BOTH………………………………………………. 3 (Go to 17g)

g. Pneumothorax size

SMALL (less than 1/3 of the hemithorax)………………… 0

MODERATE (2/3 of hemithorax)………………………… 1

MASSIVE (> 2/3 of hemithorax)……………………… 2

“.” if N/A

18. ⁭

19. ⁭

19a.

20. ⁭

21 ⁭

21a.

22. ⁭

1. Radiological Extent of Disease – See Definitions in Appendix Below

NORMAL…………………………………………….... 0

MINIMAL........................................................................ 1

MODERATELY ADVANCED………....………...…… 2

FAR ADVANCED……………………....………...…… 3

19. Radiological Impression of Parenchyma

Normal parenchyma…………………………………. 0

Lobar pneumonia………............................................. 1

Bronchopneumonia……….......................................... 2

Interstitial pneumonia………...................................... 3

Collapse/atelectasis………………………………….. 4

Fibrosis………………………………………………. 5

Fibro-cavitary pneumonia…….……………………… 6

Cavitary pneumonia………......................................... 7

Miliary TB pattern…………………………………… 8

Solitary pulmonary nodule…………………………… 9

Mass………………………………………………….. 10

Emphysema…………………………………………... 11

Pleural effusion/disease………………………………. 12

Pneumothorax ……………………………………….. 13

Pulmonary cardio-vascular disease………………….. 14

Bronchiectasis……………………………………….. 15

Others, specify………………………………………. 16 (Specify in 19a)

a. Specify Other

20. Likelihood of TB

Probable (findings consistent with TB)..……………… 0

Possible (atypical findings)…………………………… 1 1

Unlikely (findings not consistent with TB)…………… 2

Unable to assess………………....………...………….. 3

1. Radiological Diagnosis

Normal……………………..……………………..…… 0

Pulmonary tuberculosis………………………………... 1

Extra-pulmonary tuberculosis…………......................... 2

Bacterial pneumonia…………………………………… 3

PCP.........................................…………………………. 4

PKS .........................................………………………... 5

Lymphoma……………………...................................... 6

LIP…...........................................……………………… 7

OTHER.........................................……………… . 8 (Specify in 21a)

1. Specify Other __________________________________________

22. Radiologist

OKELLO/KISEMBO………………………………….. 0

KISEMBO/KAWOOYA………………………………. 1

OKELLO/KAWOOYA………………………………… 2

OTHER…………………………………………………. 8
